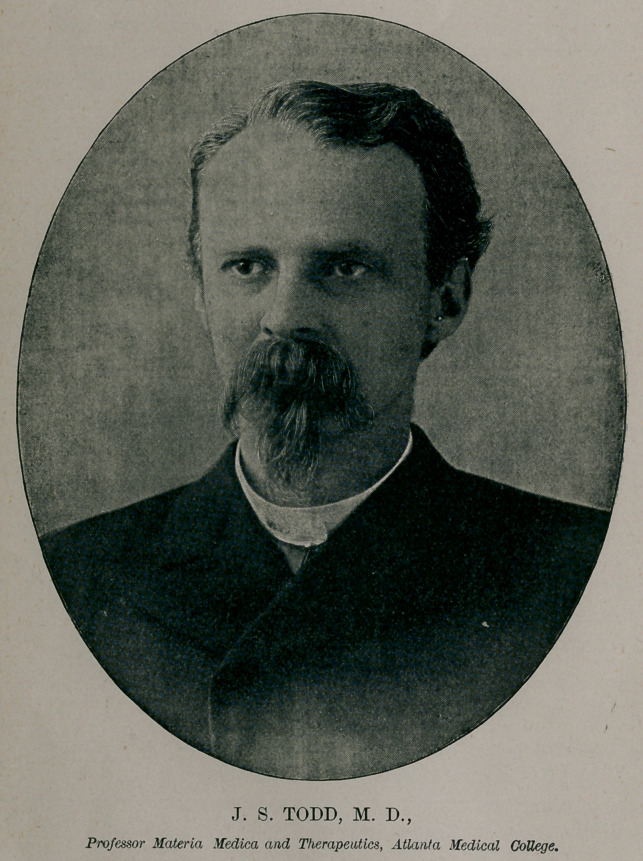# Our Portrait Gallery

**Published:** 1884-06

**Authors:** 


### OUR PORTRAIT GALLERY.

We present our readers in this number of THE JOURNAL, the portrait of one of the foremost young men in the profession in Georgia,

### DR. JAMES SCOTT TODD.

Dr. Todd was born in Alabama in 1847, where he lived until the outbreak of the late war, when he entered the army at 17 years of age in the battalion of Georgia Cadets. He was in many of the hard fought battles of the war, and was wounded a number of times. He lost an arm in one of the battles around Atlanta.

He studied medicine in West Point under Drs. Tate and Griggs, and graduated from Jefferson Medical College in Philadelphia in 1869, when he returned to West Point and practiced medicine for six years. He was elected a member of the Medical Association of Georgia in 1872. He has been Orator, and is at present one of the Censors. He is also a permanent member of the American Medical Association and was at one time assistant Secretary. He removed from West Point to Atlanta in 1875, and was very soon thereafter elected President of the Atlanta Academy of Medicine, succeeding such men as Drs. Jos. P. Logan, W. F. Westmoreland, V. H. Taliaferro, W. S. Armstrong, H. V. M. Miller and J. F. Alexander. Dr. Todd was elected Lecturer on Dermatology and proctor of the Atlanta Medical College in 1880, and in 1881 he was elected to the chair of Materia Medica and Therapeutics, a position he still fills and honors.

As a teacher, his genius is attested by the chair he now so ably fills in the Atlanta Medical College, as well as by the contributions he has made to medical literature. Among his most noted contributions may be mentioned "A Case of Opium Poisoning," *American Journal of Medical Sciences*, in which he first announced the fact that veratrum viride is an antidote to opium poisoning, "Belladonna," "Cases from Note Book," "Reports Proceedings of Atlanta Academy of Medicine, 1876–77, and '78." "Influence of the Mind over Disease," "Mercury," etc.

As a lecturer, Dr. Todd has the happy faculty of holding the attention of the class as few lecturers have. Those who have had the pleasure of hearing him will bear testimony to his rare power of imparting information, and to the esteem in which he is held by his students.

He has rapidly worked himself into the front ranks of the profession in his adopted State, and is now enjoying a lucrative practice as the result of his general information and great practical skill, and his courteous and kindly regard for every one with whom he comes in contact.

*Professor Materia Medica and Therapeutics, Atlanta Medical College.*

## Figures and Tables

**Figure f1:**